# Subcutaneous Implants of Buprenorphine-Cholesterol-Triglyceride Powder in Mice

**DOI:** 10.1155/2014/365673

**Published:** 2014-11-27

**Authors:** L. DeTolla, R. Sanchez, E. Khan, B. Tyler, M. Guarnieri

**Affiliations:** ^1^Departments of Pathology, Medicine, Epidemiology and Public Health and the Program of Comparative Medicine, School of Medicine, University of Maryland, Baltimore, MD, USA; ^2^Program of Comparative Medicine, School of Medicine, University of Maryland, Baltimore, MD, USA; ^3^Johns Hopkins School of Medicine Department of Neurological Surgery, 1550 Orleans Street CRB-264, Baltimore, MD 21231, USA

## Abstract

Subcutaneous drug implants are convenient systems for the long-term delivery of drugs in animals. Lipid carriers are logical tools because they generally allow for higher doses and low toxicity. The present study used an US Food and Drug Administration Target Animal Safety test system to evaluate the safety of a subcutaneous implant of a cholesterol-triglyceride-buprenorphine powder in 120 BALB/c mice. Mice were evaluated in 4- and 12-day trials with 1- and 5-fold doses of the intended 3 mg/kg dose of drug. One male mouse treated with three 3 mg/kg doses and surgery on days 0, 4, and 8 died on day 9. The cause of death was not determined. In the surviving 119 mice there was no evidence of skin reaction at the site of the implant. Compared to control animals treated with saline, weight measurements, clinical pathology, histopathology, and clinical observations were unremarkable. These results demonstrate that the lipid carrier is substantially safe. Cholesterol-triglyceride-drug powders may provide a valuable research tool for studies of analgesic and inflammatory drug implants in veterinary medicine.

## 1. Introduction

Guidelines for the care and use of laboratory animals uniformly recommend the use of analgesia in any procedure with a potential for pain [1]. Yet, the use of analgesics in research remains low [2, 3]. One factor that may account for the modest utilization of analgesia is the management challenge involved with intraperitoneal (IP) and subcutaneous (SC) injections of mice and rats at the 6–8-hour intervals necessary to maintain effective blood concentrations of drug [4]. In addition, it has been considered that repeated IP or SC injections in surgically traumatized rodents may induce stress responses and depress weight gain [5, 6]. Several strategies are being investigated to address this problem.

The practicality and duration of slow-release oral preparations for 11-hour morphine therapy in laboratory rats have been described [7]. Food- and water-based analgesia has been explored by several investigators [8–10]. Grant and colleagues demonstrated that liposomal morphine implants could deliver long-acting analgesia to mice [11]. More recently, Smith, Krugner-Higby, and colleagues have published a series of studies demonstrating the 2-3-day activity of lipid encapsulated morphine derivatives in laboratory animal models of pain [12–14]. Foley and colleagues described the 2-3-day efficacy of a proprietary sustained release polymer-based buprenorphine preparation in rats [15]. Carbone and coworkers described the efficacy of a similar formulation in two strains of mice [16].

In 2006, our laboratory began to investigate extended release opiate preparations for pain therapy in rodent brain and spine tumor models. We selected buprenorphine as a model drug because of the range of evidence for its safety and efficacy in laboratory medicine [17, 18]. Reports of morbidity and mortality in animal studies have been rare. Buprenorphine has a wide therapeutic index in animals. Compared to morphine, the mean effective dose (ED50) is 20-fold lower and the mean lethal dose (LD50) is significantly higher [19, 20].

Toxicity reports associated with SC cholesterol implants are rare. Lipid encapsulation generally has allowed for higher doses, decreased toxicity, and prolonged activity of opiate therapy [21, 22]. We studied a cholesterol-triglyceride-buprenorphine SC delivery system described by Pontani and Misra [23]. In a separate 4-month observational study Misra and Pontani provided initial evidence that the delivery system was safe in rats. They observed no evidence of inflammation or edema in rats implanted with 50 mg lipid-drug pellets [24].

To further validate the safety of lipid encapsulated buprenorphine, we consulted the Center for Veterinary Medicine (CVM) at the US Food and Drug Administration regarding the necessary methods for establishing the safety of new veterinary drugs. The CVM provides target animal safety (TAS) guidelines for evaluating drug toxicity in target animals [25]. Similar guidelines are used by European regulatory authorities [26]. We developed a TAS protocol including clinical observations, blood chemistry, hematology, and histopathology studies to examine the safety of a long-acting cholesterol-triglyceride-buprenorphine preparation in surgically treated mice. Studies in our laboratories have shown that the dissolution of SC cholesterol pellets depends on the location of the implant and excipients used to make the pellet [27]. We therefore examined the safety of the cholesterol-triglyceride-buprenorphine unpelleted drug powder in a SC space.

The present report describes body weight, hematology, clinical pathology, histopathology, and cage side observation measurements collected in this TAS study. These results provide additional evidence for the safety of cholesterol-based drug implants and for the safety of extended-release buprenorphine analgesia in mice.

## 2. Materials and Methods

### 2.1. Animals

TAS studies were approved by the University of Maryland Medical School Institutional Animal Care and Use Committee (IACUC). The University of Maryland, Program of Comparative Medicine, Baltimore, MD, was used for the safety study. Pharmacokinetic studies of serially collected blood samples were conducted at the Johns Hopkins University School of Medicine under a protocol approved by the Johns Hopkins IACUC. Male and female BALB/c (6–8 weeks old weighing 20–22 g) were obtained from Charles River Laboratories (Wilmington MA). The mice were inspected for general health conditions before being housed at a density of 4-5 mice per cage in 750 Lab Product cages (Lab Product, Seaford, DE) with 7087 Soft Cob bedding (Harlan, Madison, WI) and allowed free access to 2018 Teklad Irradiated Global Rodent Diet chow (Harlan, Madison, WI) and Baltimore City water. A total of 120 mice were used in the safety study. A total of 18 mice were used for pharmacokinetic study of buprenorphine blood concentrations.

### 2.2. Study Design

The study design was based on Target Animal Safety (TAS) protocol guidance to determine the safety of a generic drug [28]. The bioequivalent target range was selected from published reports demonstrating that buprenorphine blood levels greater than 0.5 ng/mL produce positive tail-flick responses in mice [29], thermal latency in dogs [30], and responses in human volunteers [31]. In a series of dose-finding studies, male and female mice were injected with increasing doses of the drug powder containing up to 25 mg/kg buprenorphine. A dose of 3 mg/kg, which afforded blood levels of more than 1 ng buprenorphine/mL for at least 2 days, was selected for the TAS study.

For the pharmacokinetic studies, mice were housed three per cage and cages were changed daily. During the TAS studies, mice were housed one per cage. The experimental unit was the cage for statistical purposes. Safety studies were conducted with 1- and 5-fold excesses of the intended dose. The study period was 4 days, 1 day more than the 3-day elimination period. Ten male and ten female mice per group were used in the first TAS study comparing a 0x (control), 1x, and 5x dose (15 mg/kg) challenge. Ten male and ten female mice per group were used in the second TAS study comparing 0x, 1x, and 5x doses repeated at three 4-day intervals. By agreement with the CVM, the control was 3 to 15 microliters of saline. Parameters evaluated in the 4- and 12-day trials included body weight, hematology, clinical chemistry, clinical observations, and gross and histopathology. According to the trial protocol, if no differences were observed in outcome measurements from animals in the control and 5x dose groups, tissues from animals in the 1x dose groups would not be further evaluated. The hypothesis tested was that the data for these parameters would be different in mice with 0x and 5x doses of cholesterol-buprenorphine drug powder.

### 2.3. Trial Structure

#### 2.3.1. Single 0, 1, and 5x Dose, 4-Day Trial

In the 4-day trial, 10 mice per group of each sex were anesthetized, subjected to a surgical procedure, and dosed on day 0 with a single control, 1x, 5x dose (15 mg/kg) of drug powder or 15 uL of saline.

#### 2.3.2. Repeat, Three 0, 1, and 5x Doses, 12-Day Trial

In the 12-day dose-repeat trial, 10 mice per group of each sex were anesthetized, subjected to a surgical procedure, and dosed with a 1x and 5x dose (15 mg/kg) of drug or 15 uL of saline on days 0, 4, and 8.

Cages were changed daily to prevent the animals from redosing by coprophagy. Mice in the single dose and repeat dose trials were evaluated by daily clinical observations for signs of distress. At the midpoint of the two trials, day 2 or day 6, half of the mice were weighed, euthanized, and then exsanguinated to collect blood for hematology and clinical chemistry. At the endpoint of each trial, day 4 of day 12, the remaining mice were euthanized to measure body weight, hematology, clinical chemistries, and histopathology.

### 2.4. Test Article Details

The cholesterol-buprenorphine drug powder was supplied by Animalgesic Laboratories Inc. (Millersville, MD). The drug powder contained USP (United States Pharmacopeia) grade buprenorphine HCl (Noramco, Wilmington, DE), cholesterol, and glycerol tristearate, (Sigma, St. Louis, MO). Drug preparations were verified for purity and content by AAI Pharma (Wilmington, NC).

### 2.5. Drug Delivery

**Figure 1:**
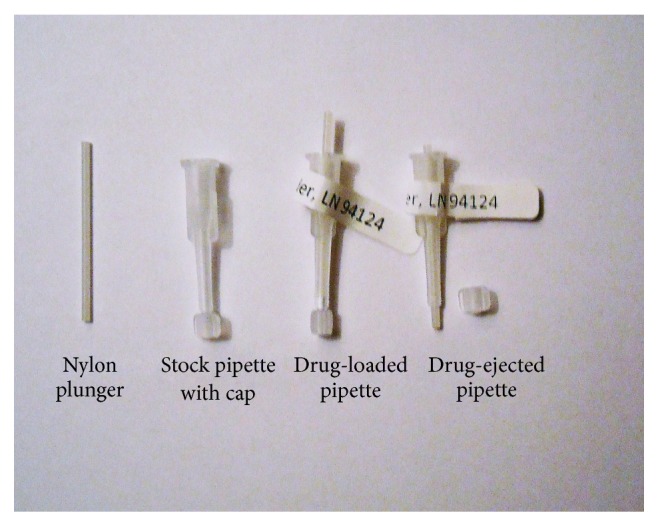
Pipets for drug powder delivery.

Dental pipets were used to deliver 3 mg aliquots of drug powder into a dorsal subcutaneous space created by a surgical procedure (described below). The drug powder was loaded into disposable 30 mm long capped dental pipets by Ora Tech (Riverton UT). The drug-filled pipets were fitted with a nylon plunger to secure the powder prior to injection in the mouse (Figure 1). At the time of surgery, the pipet cap was aseptically removed and the tip of the dental pipet was inserted into the SC space. The nylon plunger was depressed to deposit the powder into the SC space. Five drug loaded pipets were used for the 5x dose groups. To allow for histopathology evaluation of the skin at the implant site, the pipet tips were placed approximately 10 mm under the skin away from the surgical incision.

### 2.6. Buprenorphine Blood Level Measurements

Serial blood samples were obtained by facial bleeding of the superficial temporal vein [32]. Samples were taken at noon, 23–25-hour intervals after the drug was implanted. Plasma samples were used for buprenorphine measurements by ELISA. Samples of 5–20 *μ*L of plasma were analyzed in triplicate using a Buprenorphine One-step ELISA kit (International Diagnostic Systems, St. Joseph, MI). The manufacturer validated the kit for clinical drug studies with high-performance liquid chromatography-electrospray mass spectrometry (HPLC-ES-MS) procedure. All known cross-reactivities are reported by the manufacturer at <0.06%, with the exception of norbuprenorphine, which cross-reacts at 1.1%. Standards curves were prepared with five buprenorphine solutions: 0, 0.01, 0.05, 0.1, 0.5, and 1.0 ng/20 *μ*L. Absorbance was recorded at 450 nm (reference wavelength: 650 nm) using a Perkin Elmer Victor3 model 1420 microplate reader with Wallac 1420 data manager software.

### 2.7. Surgical Procedure

The surgical procedure used was based on the procedure used to implant Alzet miniosmotic pumps in mice and rats. A video of the procedure, which is briefly described below, is available at the Alzet website [33].

Each mouse was weighed prior to surgery to record baseline weights. Anesthesia was provided with isoflurane. Mice were induced with 4% isoflurane (Vetone, Boise, ID) inhalant anesthesia until deep sedation was established and then reduced to 2.5–3% dose of isoflurane maintenance. Approximately 1 cm square of mid dorsal skin was shaved and aseptically prepared by three alternating scrubs of betadine scrub and 70% isopropyl alcohol. Each mouse was transferred to a procedural table that was covered with a sterile table drape that is fluid resistant. The mouse was draped with sterile quarter drapes to outline the surgical approach site. Using sterile instruments, forceps (McCullough forceps, cross serrated jaws, 1.5 mm tip) were used to lift the aseptic skin from the lumbosacral region (dorsal aspect) and a pair of scissors (delicate operating scissors, straight, sharp-sharp, and 30 mm blade length) was used to make a 4-5 mm incision through the skin only. Bleeding, if any, was controlled with sterile gauze and light pressure. Keeping skin lifted with the forceps, a sterile pair of forceps (Halstead mosquito, straight) was used to separate the skin from the underlying muscular layer and to create approximately a 2 × 4 cm subcutaneous pocket. Using aseptic surgical techniques, the test article (1x, 5x drug powder, or saline) was injected into the lumbosacral region (dorsal aspect). The skin was then apposed using 5–0, reverse cutting, coated Vicryl sutures (Ethicon). After surgery was completed, the isoflurane was turned off and the animal was continuously monitored by the surgeon or veterinary technician until the animal's reflexes (monitored by toe pinch) returned.

All mice in the study were treated to this surgical procedure.

### 2.8. Clinical Observations

Cage conditions, motor activity, ocular findings, and the appearance of the fur were observed twice daily at about 10 am and 5 pm by a research staff. Observation forms were designed for the entry of “yes/no” scores and numerical grading of signs and symptoms including respiration, tremors, motor activity, ocular findings, nasal findings, and appearance in the morning observation period. The pm observations included ocular signs, motor activity, signs of distress, and appearance. The surgical site was observed for signs of bleeding, erythema, edema, and signs of infection: pus. Space was available for comments. The same forms were used for both TAS studies: the single 5x dose 4-day observation period and the three repeated 5x doses 12-day observation period.

### 2.9. Clinical Laboratory Tests

Blood chemistry, hematology, and histopathology were performed at the Johns Hopkins Phenotyping Core [34]. Hematology tests were performed on a Hemavet 950 Hematology System (Drew Scientific, Waterbury, CT). Values obtained included white blood cell, neutrophil, lymphocyte, monocyte, eosinophil, basophile, red blood cell, and platelet counts and hemoglobin concentration, hematocrit, mean corpuscular volume, mean corpuscular hemoglobin, mean corpuscular hemoglobin concentration, red cell distribution width, and mean platelet volume. A VetACE Clinical Chemistry system (AlfaWassermann, West Caldwell NJ) was used to measure blood chemistry profiles: cholesterol (Chol), triglycerides (Tri), uric acid (UA), total bilirubin (TBill), glucose (Glu), total protein (Tpr), calcium (Ca), urea nitrogen (BUN), creatinine (Creat), albumin (Alb), high density lipoproteins (HDL), direct bilirubin (DBill) and the enzymes creatine kinase (CK), lactic dehydrogenase (LDH), alkaline phosphatase (ALP), amylase (Amy), gamma glutamyl transferase (GGT), alanine amino transferase (ALT), and aspartate amino transferase (ASP).

### 2.10. Euthanasia

Mice were weighed and then asphyxiated with carbon dioxide followed by 1 min lack of respiration. The heart was exposed. Mice were exsanguinated via cardiac puncture to obtain approximately 0.8 mL of blood for hematology and clinical chemistry testing. Mice were then placed in 10% neutral buffered formalin.

### 2.11. Body Weights

Mice were weighed in procedure rooms with a calibrated Sartorius Acculab Precision Scale (Goetting, Germany) before they were assigned to a treatment group and within 24 hours before they were injected with one or more doses of drug powder or control suspensions on day 0. Mice scheduled for euthanasia weighed on an electronic Ohaus microbalance (Parsippany, NJ) then euthanized with carbon dioxide.

### 2.12. Histopathology

Histopathology was performed on endpoint mice listed in Table 1. These were 30 mice in the 4-day single dose and control challenges and 30 mice in the 12-day repeat dose challenges. Heart, kidneys, liver, and spleen were collected. More than 30 tissues were examined for a total of 13 slides per mouse. The tissue list is summarized in Table 2.

One male mouse in the 12-day repeat 9 mg/kg dose trial found dead on day 10 was not perfused. Because of extensive tissue autolysis and postmortem rigor, necropsy was not indicated. In the remaining mice after fixation and trimming, the tissues were processed, embedded in paraffin, sectioned, mounted on glass slides, and stained with hematoxylin and eosin (H&E). These slides were evaluated by light microscopy. Tissues listed in Table 2 were evaluated.

### 2.13. Statistics

Analyses of treatment group blood levels were made using GraphPad Prism Software Version 5.04 (La Jolla CA). Microsoft Excel v2007 was used to generate average and standard deviation (St Dev) data of hematology and clinical chemistry values.

## 3. Results

### 3.1. Blood Concentrations of Drug

As shown in Table 3, plasma concentrations of buprenorphine in mice given 3 mg/kg (1x) dose of drug averaged 45 ng/mL 6 hours after a SC drug powder implant. Approximately 2 and 6 ng of drug per mL plasma were present through day 3 in male and female mice, respectively. There was no detectable drug present by day 6 in either sex.

### 3.2. Clinical Observations

Approximately 3,300 clinical observation entries were recorded for the mice in the 4-day trial, and 9,900 entries were recorded for the mice in the 12-day trial. In the 4-day trial, 6 female and two male mice in the 1x dose group exhibited mild erythema and edema at the surgical site on day 1. These signs were not apparent on days two and three of the trial and were not seen in the subsequent 12-day trial. On average mice in the 5x dose groups showed slower movement compared to controls. They also showed more ocular findings of squinting and closed eyes. However, these movement and ocular findings did not reach statistical significance compared to controls.

One male mouse in the 12-day trial died one day following the third cycle of anesthesia, surgery, and 3 mg/kg drug powder. The carcass was not subject to necroscopy due to a clinical impression that advanced autolysis had set in. Previous observation including weight measurements provided no information to indicate distress.

### 3.3. Body Weight

In the 4-day TAS trial drug-treated female and male mice given a 3 mg/kg dose lost an average of 7% and 9% body weight by day 4, respectively. Weight losses were similar in the female and male mice given the 15 mg/kg dose, 6% and 4%, respectively. The weight losses were not significantly different from weight losses in the surgically treated control mice. Similar results were observed in the 12-day trial (Table 4).

### 3.4. Clinical Pathology

There were no differences between hematology and clinical chemistry blood values in the drug and control groups in the 4-day and the 12-day trials in the mice receiving the 1x or 5x doses of the drug powder.

### 3.5. Histopathology

The histopathology examination of the tissues from the male and female mice was unremarkable. The surgical sites appeared competent. There was no sign of infection. Because an effort was made to deposit the drug powder under the skin at least 10 mm away from the incision site, it is possible to conclude that skin above the powder was normal. In the 12-day trials where mice were injected with three 5x doses of powder at 4-day intervals for a total of 45 mg of drug powder, residual drug powder was frequently observed. There was no edema or inflammation associated the thin powder layer.

## 4. Discussion

The present report describes the morbidity and mortality encountered in a TAS trial of a SC extended release cholesterol-triglyceride-buprenorphine powder. A sensitive and specific ELISA analysis demonstrated that a single 3 mg/kg dose of drug implanted at the time of surgery afforded average plasma concentrations of drug of 45 ng/mL in 6 hours and 20 ng/mL or more for two days (Table 3). These concentrations have been consistently associated with effective pain therapy in animal and humans studies [18] and specifically with the use of buprenorphine analgesic in mice [35]. In the present study, the high concentration of drug measured 6 hours after implant indicates that the lipid carrier system rapidly released the drug. This suggests that the analgesic effects of the drug may overlap the anesthetic recovery period. Whether there is a continuum of pain management should be determined by pharmacokinetic measurements at earlier postimplant times and with the use of different anesthetics.

Pain assessments are required in TAS studies of drug implants. It is generally considered that signs of severe pain can be readily detected by experienced laboratory animal scientists, but signs of mild to moderate pain and pain that breaks through analgesia can be difficult to detect [36]. The system for the visual assessment of pain used in the present work was similar to the scoring system used by Clark and colleagues to assess pain in mice treated with liposome encapsulated oxymorphone in mice [12]. We observed no signs of severe pain. The implant appeared to be well tolerated by the mice. One death occurred in the 3 mg/kg dose group at day 9 in a male mouse that had three cycles of anesthesia and surgery in 8 days. The cause of death was not determined. There was no evidence from the clinical pathology and histopathology evaluations of the remaining male and female mice in this dose group of toxicity from the drug implant.

Several recent reports indicate that visual assessment system used in the present study to monitor pain may be limited. Mouse grimace scores appear to be highly sensitive indicators of postsurgical pain [37, 38]. Adamson and coworkers demonstrated that mouse pain scores were low when an observer was in the room. Video recording of behavior during light and dark cycles showed mild yet significant differences [12]. A separate limitation of the present study for the detection of mild pain is that subtle signs of pain can be inferred in rodents in relation to their behavior toward normal cage-mates. However, TAS studies required single housing of animals.

Drug-polymer implants have been investigated for long-term analgesia [15, 31]. Biocompatible ethylene vinyl acetate copolymers can be designed to provide weeks to months of linear drug release [39]. These polymers are removed when empty and are not optimal for routine use in laboratory medicine. Biodegradable copolymers are designed for safe drug release and adsorption. However, drug release from these polymers can be anomalous [40]. Long-term inflammatory reactions have been observed [41]. Significant skin reactions have been reported in mice and rats treated with polymer-bound buprenorphine [15, 16]. In contrast, the biodegradable lipid carrier system used in this study showed no evidence of skin toxicity, even in studies that used three implants at fivefold the intended dose. Further research is needed to determine if the cholesterol carrier is biocompatible in other strains and species and whether the absence of dermal reactions can be confirmed in long-term histopathology studies.

Buprenorphine decreases intestinal motility [19]. Nausea is commonly observed in mice and rats treated with oral or parenteral buprenorphine therapy [42]. Several investigators reported that the nausea can induce pica in rats held on corncob or hardwood bedding [43, 44]. In other reports, with appropriate husbandry, the opiate-induced nausea appears to be mild and transient [45].

All mice treated with the buprenorphine cholesterol-triglyceride drug powder lost weight. Weight loss was not significant compared to controls, and mice appeared to return to baseline weights by day 4. More research is needed to determine if the weight loss is greater in adult mice with slower growth curves.

## 5. Conclusion

The compelling needs for improved veterinary drug products support further research on chronic release drug systems. Lipid-based delivery vehicles appear to be good candidates because they are safe and biodegradable [23]. The safety profile of the extended-release cholesterol-triglyceride-buprenorphine powder described in this report confirms previous studies of long-acting preparations of micellar morphine in mice [11], liposome encapsulated oxymorphone in mice [12], and liposome-encapsulated oxymorphone in rats [13, 14]. Carbohydrate and polymer delivery systems also offer attractive candidates for further research. Regardless of the composition of the delivery system itself, one cannot assume that SC drug-bound delivery vehicles are safe. Target animal safety studies and long-term histopathology studies of the SC space are warranted for each drug-bound vehicle.

## Figures and Tables

**Table 1:** Summary of experimental design: histopathology phase.

Test or control article	Total weight administered	Number of doses	Days on test	Number of animals
Saline	0.005 mL	1	4	5♂, 5♀
Long acting drug powder	3 mg	1	4	5♂, 5♀
Long acting drug powder	15 mg	1	4	5♂, 5♀
Saline	0.015 mL	3	12	5♂, 5♀
Long acting drug powder	9 mg	3	12	5♂, 5♀
Long acting drug powder	45 mg	3	12	5♂, 5♀

**Table 2:** Tissues evaluated by microscopy.

Adrenal gland	Large intestine, colon	Small intestine, jejunum
Bone with bone marrow, femur	Liver	Small intestine, ileum
	Lung	Spinal cord with spine
Brain (cerebrum, midbrain, cerebellum, and medulla/pons)	Lymph nodes^a^	Spleen
Epididymis (males)	Mammary glands (females)	Stomach
Eyes (with optic Nerves)	Ovaries (females)	Ventral skin
Gall bladder	Pancreas	Dorsal skin surrounding implant(s)
Heart	Parathyroid gland	Testis (males)
Kidneys	Skeletal muscle, biceps femoris	Thyroid (with parathyroid)^b^
Large Intestine, cecum	Small intestine, duodenum	Urinary bladder

^a^Lymph nodes included submandibular superficial cervical collected with salivary glands from the neck; mesenteric and pancreaticoduodenal collected with mesentery and pancreas.

^b^Parathyroid glands were evaluated when present in the plane of section of the thyroid gland.

**Table 3:** Buprenorphine drug concentrations in male and female Balb/c mice, 3 mg/kg dose of buprenorphine drug powder.

Day	Male (*n* = 3)		Female (*n* = 3)	
	Average ng/mL	St Dev	Average ng/mL	St Dev
0	0.0	0.0	0.0	0.0
0.25	45.2	0.5	44.9	3.7
1	42.9	6.6	41.6	3.7
2	20.8	5.8	27.2	3.2
3	2.1	2.1	5.8	3.2
4	0.0	0.0	2.8	3.4
6	0.0	0.0	0.0	0.0

**Table 4:** Weight change in male and female mice, 3 and 15 mg/kg dose of buprenorphine drug powder.

Day	Female mice (*n* = 5)			Male mice (*n* = 5)		
	Control	1x dose	5x dose	Control	1x dose	5x dose
	Avg ± St Dev	Avg ± St Dev	Avg ± St Dev	Avg ± St Dev	Avg ± St Dev	Avg ± St Dev
0	18.0 ± 0.5	17.9 ± 0.4	18.6 ± 0.7	21.3 ± 1.0	21.7 ± 0.9	19.3 ± 1.0
4	17.6 ± 0.8	16.6 ± 0.5	17.5 ± 0.5	21.4 ± 1.3	19.8 ± 2.3	18.6 ± 1.2
